# Effects of mucopolysaccharide polysulphate on tight junction barrier in human epidermal keratinocytes

**DOI:** 10.1111/exd.14637

**Published:** 2022-07-11

**Authors:** Mika Fujikawa, Hiroko Sugimoto, Rie Tamura, Koki Fujikawa, Ami Yamagishi, Yuhki Ueda

**Affiliations:** ^1^ Kyoto R&D Center, Maruho Co., Ltd. Kyoto Japan

**Keywords:** claudin‐1, heparinoid, mucopolysaccharide polysulphate, skin barrier, tight junction

## Abstract

Tight junctions (TJs) play important roles in epidermal barrier function and their dysfunction is involved in the pathogenesis of various skin diseases, including atopic dermatitis (AD). Mucopolysaccharide polysulphate (MPS) is the active ingredient of a moisturizing agent used to treat xerosis in patients with AD; however, its mechanism of action on TJ barrier function remains unclear. To elucidate the effects of MPS on TJs, adult human epidermal keratinocyte (HEKa) cells were exposed to MPS, subjected to Western blotting and quantitative PCR analyses for the investigation of TJ‐related factors. MPS treatment significantly increased the mRNA and protein expression of claudin‐1 (CLDN1) and zonula occludens‐1, and significantly increased transepithelial electrical resistance (TEER), which indicates TJ integrity. Conversely, the sulphated and non‐sulphated glycosaminoglycans, chondroitin sulphate and hyaluronic acid, respectively, had little effect on TEER or the expression of mRNAs or TJ‐related proteins. Interestingly, MPS treatment also inactivated the extracellular signal‐regulated kinase signalling pathway, which is known to negatively regulate CLDN1 expression. Furthermore, MPS notably improved the reduction in CLDN1 expression and TEER caused by histamine, which is upregulated in the skin of patients with AD and is known to disrupt the TJ barrier function. Taken together, these findings demonstrate that treatment with the moisturizing agent, MPS, can repair TJ dysfunction and could therefore represent a new therapeutic option for treating patients with AD.

## 1 INTRODUCTION

The skin is the only epithelial surface with two barrier structures, the stratum corneum (SC) and tight junctions (TJs),^1^, ^2^ which interact to prevent the penetration of external antigens as well as the leakage of internal constituents.^3^ The SC constitutes the outermost layer of the epidermis, whereas TJs form intercellular junctions in the second layer of the stratum granulosum and create a barrier against water, ions, and macromolecules.^4^ TJs contain multiple different components, including transmembrane proteins (claudin (CLDN), occludin (Ocln)), junctional adhesion molecules and TJ plaque proteins (zonula occludens [ZO] proteins, multi‐PDZ domain protein‐1 and cingulin).^4^, ^5^ Recent studies have revealed that various TJ components are dynamically regulated by environmental factors to modulate their functions.

Claudin‐1 (CLDN1) is a major component of TJs that is localized in the basal and suprabasal layers of the epidermis.^6^, ^7^ The overexpression of CLDN1 in Madin–Darby canine kidney epithelial cells has been reported to significantly increase transepithelial electrical resistance (TEER), which indicates TJ integrity in epithelial or endothelial cells.^8^ Similarly, silencing CLDN1 expression in human skin keratinocytes was found to decrease TEER^9^ and CLDN1 knockout mice display TJ barrier deterioration as well as abnormal SC formation and SC barrier defects.^6^ The TJ barrier in the stratum granulosum, therefore, plays a crucial role in the complete SC formation and barrier function.^10^

In humans, CLDN1 mutations are associated with ichthyotic, xerotic and flaky skin, as well as the development of skin diseases such as atopic dermatitis (AD), psoriasis and neonatal ichthyosis‐sclerosing cholangitis syndrome.^11^ AD is characterized by a hyperreactive immune response to allergens and dry skin, caused partly by epidermal barrier dysfunction,^12^, ^13^ which promotes immunological responses by allowing the invasion of allergens/antigens and microbes.^12^ Some studies have reported that CLDN1 expression is significantly decreased in both lesional and non‐lesional skin samples from patients with AD,^14^, ^15^ and that single nucleotide polymorphisms in CLDN1 are associated with AD.^14^ Furthermore, CLDN1 in skin is downregulated with age,^16^ suggesting that TJ dysregulation could play a role in the pathogenesis of AD in elderly patients.^13^ Since keratinocyte TJ dysfunction plays an important role in the pathogenesis of AD, it could represent a potential therapeutic target.

Patients with AD are treated using topical corticosteroids and moisturizers which can effectively maintain the remission of eczema lesions and are relatively safe. Prompt treatment with remission induction therapy can also reduce inflammation and itching to maintain remission alongside moisturizers.^17^ Mucopolysaccharide polysulphate (MPS), which consists of polysulphated chondroitin sulphate (ChS), is an active ingredient in Hirudoid®, a moisturizer prescribed to treat xerosis and other dermatological diseases associated with dry skin in Japan. The repeated topical application of an MPS‐containing cream was reported to improve SC hydration and epidermal barrier function by upregulating the mRNA expression of differentiation marker proteins, lipid synthesis enzymes, keratinocyte proliferation and antimicrobial peptides.^18^ In addition, treatment with MPS‐containing ointment was able to restore impaired SC barrier function and decreased SC hydration in a guinea pig of dry skin model.^19^, ^20^ Although MPS is known to improve SC barrier function, its effects on TJs remain poorly understood.

Here, we investigated the specific effects of MPS on the TJ barrier in adult human epidermal keratinocyte (HEKa) cells compared with the sulphated and non‐sulphated glycosaminoglycans (GAGs), ChS and hyaluronic acid (HA), respectively. In addition, we examined the effect of MPS on TJ impairment in an in vitro model of AD induced by histamine addition.^21^, ^22^ Together, the findings in this study suggest that MPS may represent a new therapeutic option for treating patients with AD.

## 2 METHODS

### 2.1 Cell culture and reagents

HEKa cells (Thermo Fisher Scientific) were maintained in HuMedia‐KG2 (Kurabo Industries) containing calcium (0.15 mM), human epidermal growth factor (0.1 ng/ml), insulin (10 μg/ml), hydrocortisone (0.67 μg/ml), gentamicin (50 μg/ml), amphotericin B (50 ng/ml) and bovine brain pituitary extract (0.4% v/v) at 37°C in a humidified atmosphere containing 5% CO_2_. HEKa cells were seeded on cell culture plates at a density of 125 000 cells/cm^2^ to ensure confluency when compounds were added and subsequently incubated for 6 h. Cells were treated with or without MPS, ChS (Maruho), HA (molecular weight: 16 kDa; Tokyo Chemical Industry) or histamine (Tokyo Chemical Industry) in HuMedia‐KG2 for a maximum of 48 h at 37°C. Control keratinocytes were treated with Humedia‐KG2 only (vehicle control).

### 2.2 Quantitative reverse transcriptase‐polymerase chain reaction (qRT‐PCR)

HEKa cells cultured with or without MPS, ChS or HA were washed with Dulbecco's phosphate‐buffered saline (Sigma‐Aldrich) and total RNA was extracted using a QIA shredder and a RNeasy Mini Kit (Qiagen, Valencia, CA, USA) according to the manufacturer's instructions. cDNA was prepared using a High‐Capacity cDNA Reverse Transcription Kit (Thermo Fisher Scientific). qRT‐PCR was performed using TaqMan Gene Expression Master Mix (Thermo Fisher Scientific), TaqMan Gene Expression Assays (Thermo Fisher Scientific) and TaqMan primer/probes on a QuantStudio 7 Flex Real‐Time PCR System (Thermo Fisher Scientific), with standard settings. The following TaqMan primer/probes (Thermo Fisher Scientific) were used: *claudin‐1* (*CLDN1*: Hs00221623_m1), *zonula occludens‐1* (*ZO‐1*: Hs01551861_m1) and the housekeeping gene *glyceraldehyde‐3‐phosphate dehydrogenase* (*GAPDH*; Hs99999905_m1). The comparative cycle time method was used to determine the expression of target genes normalized to *GAPDH* expression.

### 2.3 Western blot analysis

HEKa cells were washed with Tris‐buffered saline (TBS, Nacalai Tesque) and lysed in radioimmunoprecipitation assay buffer (Nacalai Tesque) containing protease inhibitors (Millipore) and phosphatase inhibitors (Thermo Fisher Scientific). Equal amounts of total protein quantified using the Pierce BCA Protein Assay were separated using sodium dodecyl sulphate‐polyacrylamide gel electrophoresis and transferred to a polyvinylidene fluoride membrane (Bio‐Rad Laboratories, CA, USA). After blocking for 1 h with 5% skim milk (Nacalai Tesque) or 0.5% bovine serum albumin (BSA) solution (Nacalai Tesque), the membranes were incubated with the antibodies listed in Table 1, with β‐actin as a loading control. Signals were detected using SuperSignal™ West Dura Extended Duration Substrate (Thermo Fisher Scientific) with a Lumino Imager (LAS‐4000 mini, Fujifilm).

**TABLE 1:** Antibodies used for Western blotting and immunofluorescence

Application	Antibody	Dilution	Brand
Western blotting	CLDN1	1/5000	Thermo Fisher Scientific
	Phospho‐p44/42 MAPK (ERK1/2)	1/10000	Cell Signalling
	p44/42 MAPK (ERK1/2)	1/10000	Cell Signalling
	ZO‐1	1/2000	Invitrogen
	β‐actin	1/5000	Cell Signalling
	Anti‐rabbit IgG‐HRP	1/10000	Cell Signalling
	Anti‐mouse IgG‐HRP	1/10000	Cell Signalling
Immunofluorescence	CLDN1	1/1000	Abcam
	Anti‐rabbit IgG Alexa Fluor® 488	1/400	Thermo Fisher Scientific

### 2.4 Immunofluorescence

HEKa cells grown to confluence on chamber slides (Watson) were fixed with 4% paraformaldehyde (FUJIFILM Wako Pure Chemical, Osaka, Japan), blocked using Blocking One (Nacalai Tesque) and incubated with the antibodies listed in Table 1. After staining with ProLong™ Diamond Antifade Mountant containing 4′,6‐diamidino‐2‐phenylindole (DAPI, Thermo Fisher Scientific), photomicrographs were obtained using a confocal laser scanning microscopy (FLUOVIEW FV3000 instrument, Olympus).

### 2.5 TEER

The barrier function of HEKa cells was assessed by measuring the resistance of a cell‐covered electrode using an ECIS‐Zθ instrument (Applied Biophysics) as described previously.^23^ The change in TEER value after the addition of the test agent (0 h) was measured at a low frequency (4 kHz) for 48 consecutive hours.

### 2.6 Statistical analysis

Data were expressed as the mean ± standard error (SE). Significant differences between two groups were evaluated using an F‐test followed by the Student's *t*‐test or Aspin‐Welch's *t*‐test. Significant differences between three or more groups were evaluated using Dunnett's multiple comparison test. Pairwise multiple comparisons were performed using Tukey's test. All statistical analyses were performed using EXSUS software (CAC Croit Corporation). Statistical significance was set at *p* < 0.05.

## 3 RESULTS

### 3.1 Effects of MPS, ChS and HA on CLDN1 and ZO‐1 expression in HEKa cells

**FIGURE 1:**
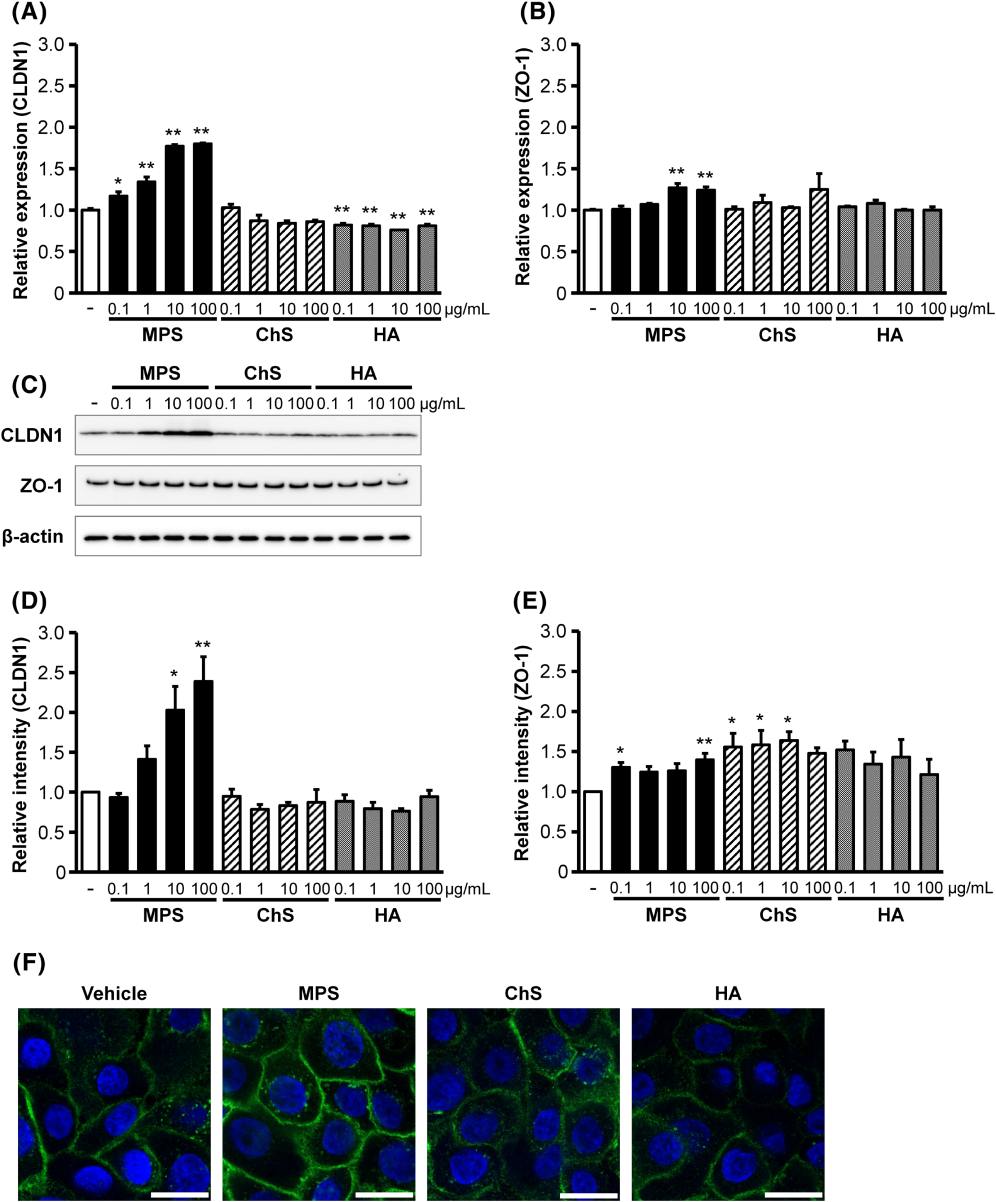
Effects of MPS, ChS and HA on CLDN1 and ZO‐1 expression in HEKa cells. HEKa cells were cultured with or without MPS (0.1, 1, 10 and 100 μg/ml), ChS (0.1, 1, 10 and 100 μg/ml) or HA (0.1, 1, 10 and 100 μg/ml) for 48 h. CLDN1 (A) and ZO‐1 (B) mRNA expression was measured using real‐time RT‐PCR. CLDN1 and ZO‐1 proteins expression in whole‐cell extracts was analysed using Western blotting with respective antibodies (C) and normalized to β‐actin (D, E). Data represents the mean ± SE (*n* = 3). *p < 0.05, **p < 0.01 versus vehicle (Dunnett's multiple comparison test). (F) HEKa cells were cultured with HuMedia‐KG2 medium containing MPS, ChS or HA (100 μg/ml) for 48 h. CLDN1 was detected using specific anti‐CLDN1 antibodies. Scale bars represent 20 μm. ChS, chondroitin sulphate; CLDN1, claudin‐1; HA, hyaluronic acid; HEKa, adult human epidermal keratinocytes; MPS, mucopolysaccharide polysulphate; ZO‐1, zonula occludens‐1

First, we evaluated the effects of MPS on the expression of the TJ barrier‐related proteins, CLDN1 and ZO‐1, in HEKa cells. Compared with the vehicle control, MPS (≥0.1 μg/ml) significantly increased CLDN1 mRNA expression in a concentration‐dependent manner. Conversely, neither ChS nor HA were able to increase CLDN1 mRNA expression at 100 μg/ml and HA significantly decreased CLDN1 mRNA expression at all concentrations (Figure 1A). ZO‐1 mRNA expression was significantly increased by 10 and 100 μg/ml MPS but was not significantly altered by ChS or HA (Figure 1B). CLDN1 and ZO‐1 proteins expression were also confirmed in HEKa cells treated with MPS, ChS, or HA (Figure 1C). Although MPS (≥10 μg/ml) significantly increased CLDN1 protein expression in a concentration‐dependent manner, ChS and HA had little effect on CLDN1 protein levels (Figure 1D). Moreover, ZO‐1 protein levels increased in all groups treated with MPS, ChS and HA, and were significantly higher in some groups (MPS 0.1, 100 μg/ml, ChS 0.1, 1, and 10 μg/ml; Figure 1E), but not in a concentration‐dependent manner. CLDN1 immunostaining 48 h after the addition of MPS, ChS and HA revealed that MPS strongly increased CLDN1 expression on the cell membrane compared with the vehicle, ChS and HA (Figure 1F), particularly along cell–cell borders. Together, these findings suggest that MPS can increase CLDN1 mRNA and protein expression in normal human keratinocytes.

### 3.2 Effects of MPS, ChS and HA on TEER in HEKa cells

**FIGURE 2:**
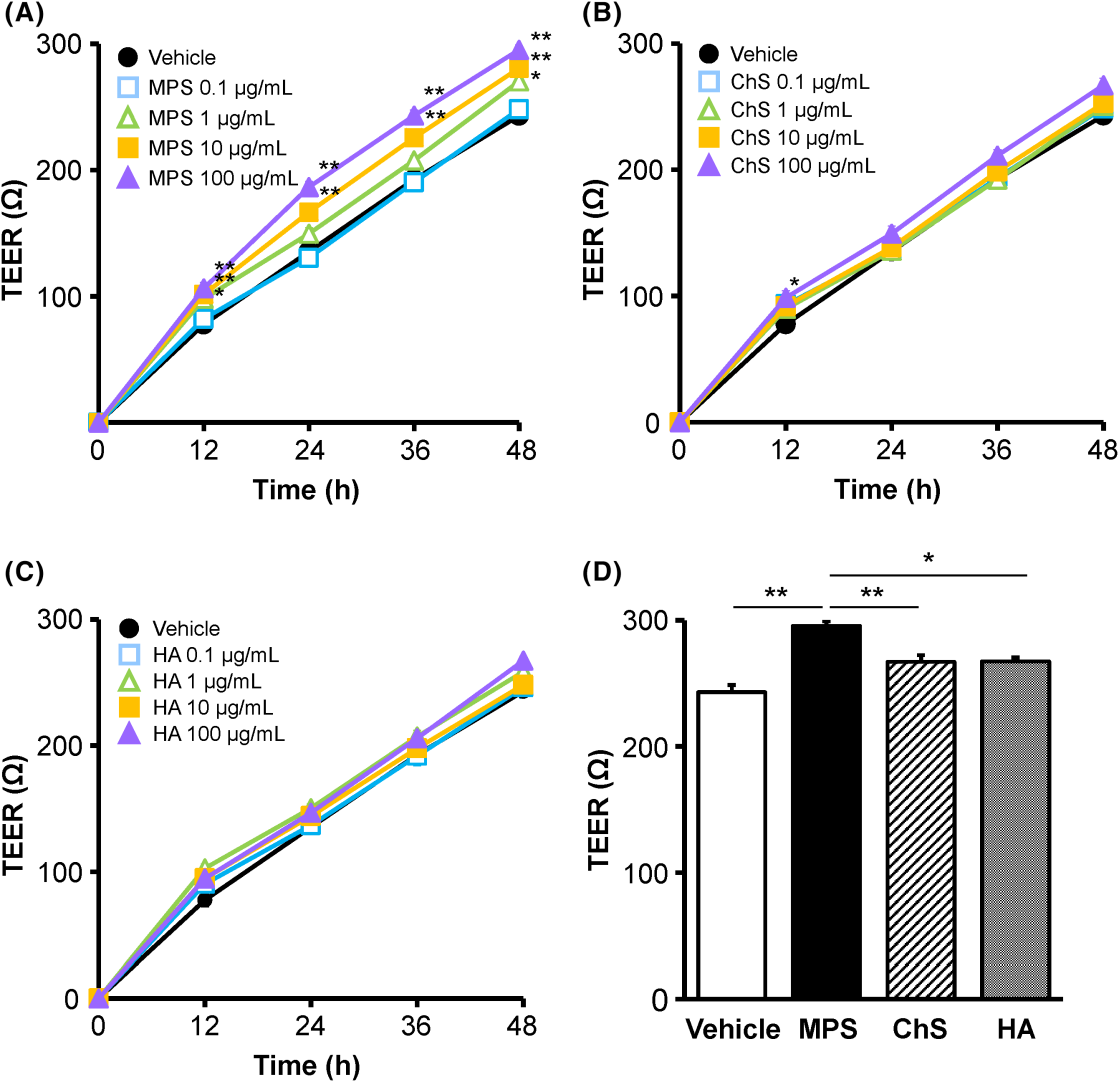
Effects of MPS, ChS and HA on TEER in HEKa cells. (A–C) TEER was measured for 48 h after HEKa cells had been treated with or without, MPS (0.1, 1, 10 and 100 μg/ml), ChS (0.1, 1, 10 and 100 μg/ml) or HA (0.1, 1, 10 and 100 μg/ml). Data represent the mean ± SE (*n* = 3). *p < 0.05, **p < 0.01 versus vehicle (Dunnett's multiple comparison test). (D) TEER (48 h) of cells treated with or without MPS, ChS or HA (100 μg/ml). *p < 0.05, **p < 0.01 vs vehicle or 100 μg/ml MPS (Tukey's test). ChS, chondroitin sulphate; HA, hyaluronic acid; HEKa, adult human epidermal keratinocyte; MPS, mucopolysaccharide polysulphate; TEER, transepithelial electrical resistance

To further investigate the effects of MPS, ChS and HA on TJ barrier function, we measured TEER, which has been associated with CLDN1 and ZO‐1 expressions in several cell types, including keratinocytes.^14^, ^24^ Notably, TEER increased significantly in a concentration‐dependent manner in cells treated with 1–100 μg/ml MPS compared with the vehicle control up to 48 h after addition, whereas ChS and HA did not increase the TEER except at 12 h at 100 μg/ml ChS (Figure 2A–C). In addition, the TEER values of cells treated with 100 μg/ml MPS were significantly higher after 48 h than in cells treated with 100 μg/ml ChS or HA (Figure 2D). Since MPS increased the TEER of keratinocytes, we hypothesized that MPS may promote TJ barrier formation.

### 3.3 Mechanisms underlying the effects of MPS on the barrier function of HEKa cells

**FIGURE 3:**
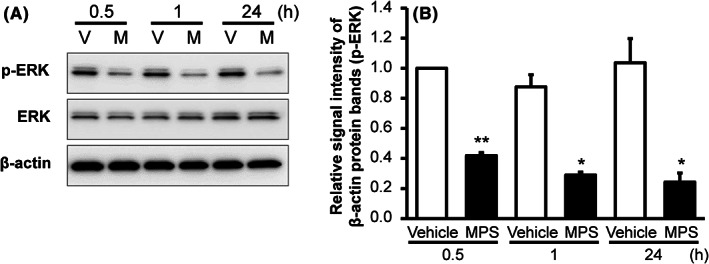
Effects of MPS on ERK phosphorylation in HEKa cells. (A) HEKa cells were cultured with or without MPS (100 μg/ml) for 0.5–24 h. p‐ERK and ERK expression were measured in whole‐cell extracts using Western blotting with the respective antibody. V: vehicle, M: MPS. (B) p‐ERK band intensity was quantified and normalized to β‐actin. Data represent the mean ± SE (*n* = 3). *p < 0.05, **p < 0.01 versus vehicle (Student's *t*‐test). HEKa, adult human epidermal keratinocytes; MPS, mucopolysaccharide polysulphate

Next, we investigated the signalling pathways through which MPS activates CLDN1 and enhances epidermal barrier function. Previous studies have reported that ERK signalling is involved in the barrier function of keratinocytes.^23^ Therefore, we measured the protein levels of total and phosphorylated ERK (p‐ERK) in cells treated with 100 μg/ml MPS. Although total ERK levels remained unchanged, p‐ERK expression was inhibited by MPS treatment from 0.5 to 24 h (Figure 3A,B). Thus, MPS may suppress ERK signalling in order to improve epidermal barrier function.

### 3.4 Effects of MPS, ChS and HA on histamine‐induced CLDN1 and TEER inhibition in HEKa cells

**FIGURE 4:**
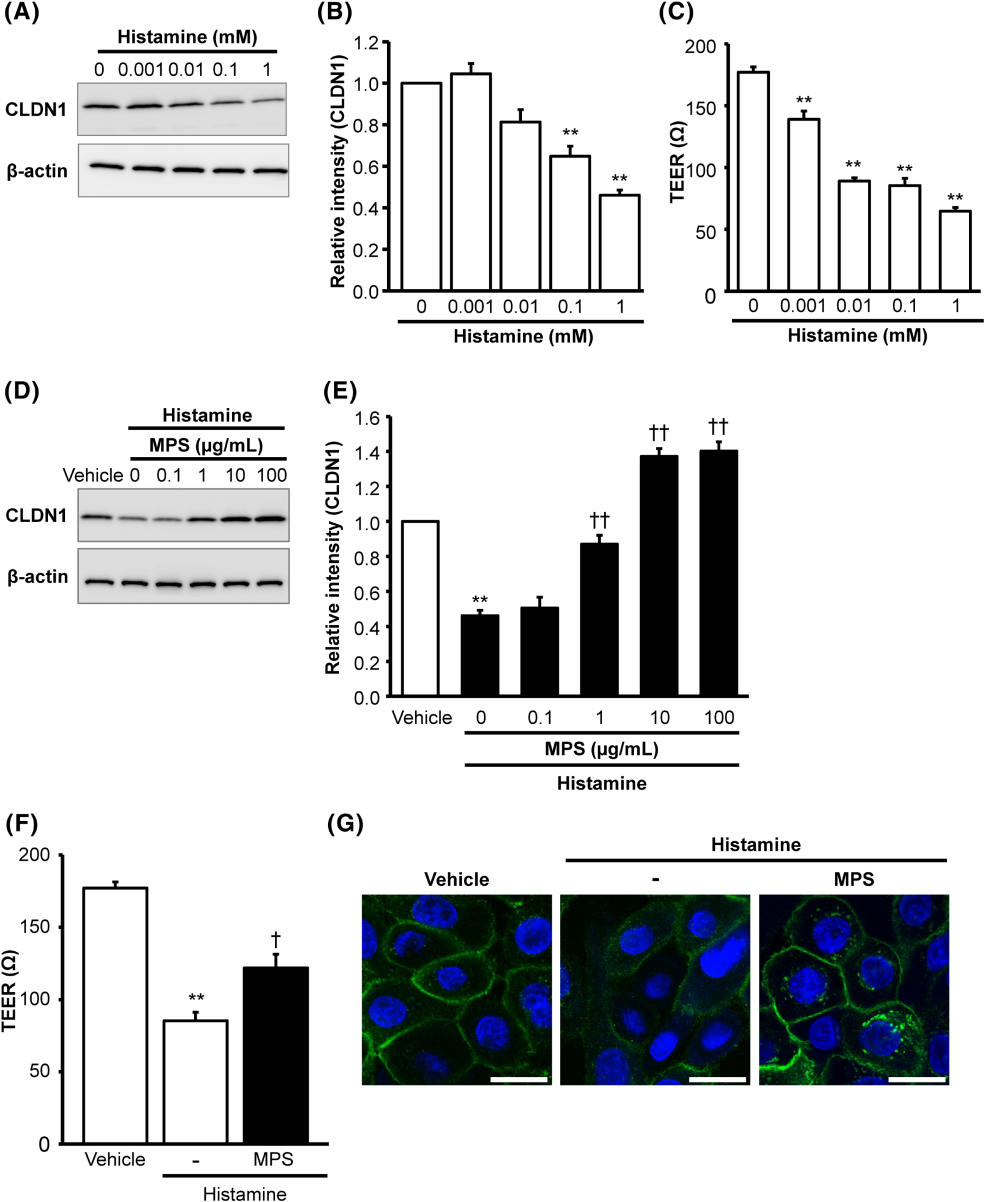
Effects of MPS on reduced CLDN1 expression and TEER induced by histamine in HEKa cells. HEKa cells were cultured with or without histamine (0.001, 0.01, 0.1 and 1 mM) for 48 h. (A, B) CLDN1 expression in whole‐cell extracts was analysed using Western blotting with appropriate antibodies. Data represent the mean ± SE (*n* = 4). ***p < 0.01 versus vehicle (Dunnett's multiple comparison test). (C) TEER (48 h) in cells treated with or without the vehicle and histamine (0.001, 0.01, 0.1 or 1 mM). Data represent the mean ± SE (*n* = 3). **p < 0.01 versus vehicle (Dunnett's multiple comparison test). (D, E) HEKa cells were cultured with or without histamine (0.1 mM) and MPS (0.1, 1, 10, and 100 μg/ml) for 48 h. CLDN1 expression was detected in whole‐cell extracts using Western blotting with appropriate antibodies. Data represent the mean ± SE (*n* = 3). **p < 0.01 versus vehicle (Aspin‐Welch's *t*‐test). ^††^ *p* < 0.01 versus histamine (0.1 mM) (Dunnett's multiple comparison test). (F) TEER (48 h) of HEKa cells were cultured with or without histamine (0.1 mM) and MPS (100 μg/ml). Data represent the mean ± SE (*n* = 3). **p < 0.01 versus vehicle (Student's *t*‐test). ^†^ *p* < 0.05, versus histamine (0.1 mM) (Student's *t*‐test). (G) HEKa cells were cultured with or without histamine (0.1 mM) and MPS (100 μg/ml) for 48 h. CLDN1 expression was detected using specific anti‐CLDN1 antibodies. Scale bars represent 20 μm. ChS, chondroitin sulphate; CLDN1, claudin‐1; HA, hyaluronic acid; HEKa, adult human epidermal keratinocytes; MPS, mucopolysaccharide polysulphate; TEER, transepithelial electrical resistance

Since histamine has been reported to inhibit the expression of TJ‐related proteins, such as CLDN1, claudin‐4 and Ocln in organotypic skin models,^21^ we examined the effects of MPS, ChS and HA in histamine‐treated cells. First, we determined the optimal concentration of histamine in HEKa cells by measuring the expression of CLDN1 and TEER. The addition of 0.1–1 mM histamine for 48 h significantly decreased CLDN1 protein expression in a concentration‐dependent manner (Figure 4A,B). Similarly, histamine significantly decreased TEER in a concentration‐dependent manner at concentrations as low as 0.001 mM (Figure 4C). Therefore, we selected a 0.1 mM histamine as the optimal concentration to investigate cotreatment with MPS, ChS or HA. As shown in Figure 4D,E, MPS restored the decrease in CLDN1 expression induced by histamine in a concentration‐dependent manner, but ChS and HA did not (data not shown). Similarly, 100 μg/ml MPS significantly restored the histamine‐induced decrease in TEER (Figure 4F) while ChS or HA was unable to exert any restorative effects (data not shown). Fluorescence immunostaining confirmed that histamine decreased CLDN1 expression in HEKa cells and that 100 μg/ml MPS could reverse this inhibitory effect (Figure 4G). These results suggest that MPS can improve TJ barrier disruption induced by histamine, which is found at higher levels in the skin affected by inflammatory conditions such as AD.

## 4 DISCUSSION

TJs play important roles in epidermal barrier function and their impairment is involved in the pathogenesis of various skin diseases, such as AD. MPS is the active ingredient of a moisturizing agent used to treat xerosis in patients with AD; however, its mechanism of action on TJ barrier function remains unclear. In this study, we investigated the effect of MPS on the TJ barrier in HEKa cells, as well as the underlying mechanism. We found that MPS increased CLDN1 and ZO‐1 mRNA and protein expressions, while increasing the TEER, suggesting that MPS may enhance TJ barrier function. In particular, MPS significantly increased CLDN1 expression, indicating that the increase in TEER induced by MPS may be more strongly associated with its effects on CLDN1. Bergmann et al. previously reported that TEER is highly correlated with the degree of CLDN1 expression in epidermal keratinocytes.^9^ In patients with AD, decreased CLDN1 expression is known to result in TJ and epidermal barrier dysfunction, leading to inflammatory changes in human epidermis. Together with our findings, these studies suggest that CLDN1 plays an important role in the pathogenesis of diseases involving TJ barrier disruption and may be closely related to the improvement of TJ barrier function induced by MPS.

Although we found that MPS enhanced CLDN1 expression and TEER, GAGs such as ChS and HA, which are widely distributed in various organs and skin,^25^ were unable to exert these effects. Chondroitin 6‐sulphate and 4‐sulphate, which are the main isoforms of ChS and HA, are synthesized in human epidermal keratinocytes^26^, ^27^; however, their direct effects on TJ barrier function in keratinocytes have not yet been fully elucidated. Chondroitin 6‐sulphate was recently associated with skin barrier function in 6‐O‐sulpho‐transferase‐1 knockout mice. 6‐O‐sulpho‐transferase‐1 is involved in the biosynthesis of chondroitin 6‐sulphate.^28^ Notably, the knockout mice displayed impaired epidermal permeability and a decrease in the expression of skin barrier‐related proteins, including CLDN1. In addition, HA has been reported to enhance TJ barrier function and upregulate the mRNA and protein expression of TJ‐related proteins in reconstructed human epidermis.^29^, ^30^ Although these previous studies suggested that ChS and HA may regulate TJ barrier function, they had little effect on TJ barrier function in this study. This discrepancy may be due to the different molecular weights of the GAGs used in our experiments. For instance, the molecular weight of HA in our study was 16 kDa, whereas previous studies have detected that, at a low molecular weight of 50 kDa, HA has a greater positive effect on the TJ barrier by upregulating TJ‐related genes than at 800 kDa.^29^ The binding affinity of GAGs is determined by their charge density due to ionic interactions between the highly acidic sulphate groups and basic side chains of arginine and lysine, as well as structural features.^31^, ^32^, ^33^ Since MPS consists of polysulphated ChS, it has more negative charges due to sulphation than ChS or HA, suggesting that polysulphation is important for enhancing TJ barrier function. Moreover, these negatively charged groups could allow MPS to interact more strongly with proteins, including ligands and cell surface receptors, than HA or ChS, and more effectively enhance the expression of TJ barrier‐related proteins.

In this study, we found that histamine decreased CLDN1 expression and TEER in HEKa cells, consistent with previous reports in which histamine was shown to promote keratinocyte proliferation and inhibit their differentiation by disrupting TJ integrity, inhibiting CLDN1 expression, and reducing TEER.^21^, ^22^ In addition to disrupting TJ barrier function in keratinocytes, histamine has been reported to be upregulated in lesional and non‐lesional skin samples from patients with AD.^34^ The skin of patients with AD also contains increased numbers of mast cells, which release histamine, even before the onset of inflammation.^35^ The prolonged exposure of normal skin to reduced environmental humidity has also been shown to increase the number of mast cells and the histamine content in the dermis.^36^ In our study, TJ barrier impairment was observed after treatment with histamine concentrations over 0.1 mM, which corresponds to the concentrations that can be reached after mast cell degranulation (0.01–1 mM).^37^ In human skin models, histamine has been reported to not only reduce the expression of protein markers of differentiation, but also that of TJ junction proteins in human skin models.^21^ Furthermore, topical histamine receptor antagonists have been shown to inhibit inflammation in dermatitis, suggesting that they may contribute toward improving epidermal barrier homeostasis by increasing the expression of differentiation marker proteins and renewing the supply of intercellular lipids in lamellar granules.^38^ Thus, histamine may reduce barrier function in diseases accompanied by an increase in mast cells, such as AD. Consistently, we found that MPS improved histamine‐induced TJ barrier impairment, which was similar to that observed in AD. MPS may, therefore, partially improve TJ barrier function by increasing the expression of CLDN1, even in patients with TJ barrier impairment due to histamine‐induced inflammatory responses in AD.

To clarify the specific mechanism through which MPS enhanced TJ barrier function, we examined the effect of MPS on pathways involved in TJ barrier regulation. Since histamine is known to reduce TJ barrier function via ERK signalling in epithelial cells,^39^ we speculated that MPS may improve TJ barrier function via the ERK signalling pathway, which regulates important biological processes such as proliferation and differentiation.^40^ ERK signalling pathway activation has also been associated with barrier disruption in epithelial cells and reduced TJ protein expression in keratinocytes. For instance, ERK1/2 activation causes TJ disruption in human corneal epithelial cells^41^ and ERK inhibition induces keratinocyte differentiation.^42^ Interleukine‐33, a cytokine associated with TJ barrier disruption in AD through the ERK/STAT3 pathway, has been reported to downregulate CLDN1, leading to TJ barrier disruption in keratinocytes.^43^ Since ERK signalling is involved in changes in CLDN1 expression and TJ barrier function in keratinocytes, we hypothesized that MPS may improve TJ barrier function and histamine‐induced TJ barrier disruption in HEKa cells via the ERK pathway. Histamine signalling via the H1 receptor in keratinocytes also regulates TJ opening by activating ERK1/2 in human nasal epithelial cells,^39^ suggesting that histamine promotes TJ destruction by activating ERK1/2 in keratinocytes. Here, we demonstrated that MPS enhances TJ barrier function in HEKa cells and inhibits histamine‐induced TJ barrier impairment. Additionally, MPS persistently suppressed ERK phosphorylation in HEKa cells for up to 24 h. Therefore, the beneficial effects of MPS on TJ barrier function in both normal and histamine‐treated cells may be linked to the suppression of ERK signalling.

Despite its novel findings, this study had some limitations. Firstly, we were unable to fully elucidate the mechanisms through which MPS affects TJ barrier, as this study was based solely on in vitro data. The repeated application of topical agents containing MPS has been reported to increase the expression of differentiation markers and enhance barrier recovery in mice.^18^ Therefore, future studies should be conducted to evaluate the effect of topical MPS‐containing products on the expression of TJ barrier‐related proteins and TJ barrier function in vivo. In addition, to clarify whether the TJ barrier enhancement of MPS simply promotes cell differentiation, we need to investigate the effect of MPS on the expression of other differentiation markers such as filaggrin and loricrin in keratinocytes. Indeed, both the TJ barrier and keratinocyte differentiation are closely and complementarily related in the maintenance of the skin barrier,^44^, ^45^ suggesting that MPS may also promote differentiation in keratinocytes, as found in in vivo studies.^18^ Furthermore, we were unable to evaluate the effects of MPS on other claudins or other proteins involved in TJ formation in keratinocytes, or the specific mechanism underlying its suppression of ERK phosphorylation. Thus, future studies should investigate the upstream proteins that suppress ERK signalling, as well as the importance of ERK signalling inhibition in barrier recovery induced by MPS. Moreover, carbohydrate recognition mechanisms such as CD44 signalling involved in TJ barrier^44^ should be also investigated.

In conclusion, this study demonstrated that MPS increases the expression of CLDN1 and ZO‐1, enhances TJ barrier function and promotes the recovery of keratinocytes following histamine‐induced TJ impairment. Together, these findings suggest that MPS could prevent the onset and exacerbation of various skin diseases, such as AD and skin aging, by recovering the loss of TJ barrier function.

## AUTHOR CONTRIBUTIONS

MF and HS designed the study, performed the experiments, analysed the data and wrote the manuscript. RT, KF and AY performed experiments and reviewed the manuscript. YU designed the study and wrote the manuscript. All authors read and approved the final manuscript.

## CONFLICT OF INTEREST

All authors are employees of Maruho Co., Ltd.

## Data Availability

The data that support the findings of this study are available from the corresponding author upon reasonable request.
